# Overexpression of Peptide-Encoding *OsCEP6.1* Results in Pleiotropic Effects on Growth in Rice (*O. sativa*)

**DOI:** 10.3389/fpls.2016.00228

**Published:** 2016-03-02

**Authors:** Zhipeng Sui, Tianya Wang, Hongjian Li, Ming Zhang, Yangyang Li, Ruibin Xu, Guofang Xing, Zhongfu Ni, Mingming Xin

**Affiliations:** ^1^Key Laboratory of Crop Heterosis and Utilization (MOE) and State Key Laboratory for Agrobiotechnology, Beijing Key Laboratory of Crop Genetic Improvement, China Agricultural UniversityBeijing, China; ^2^National Center for Plant Gene Research–BeijingBeijing, China; ^3^Qingdao Agricultural UniversityQingdao, China; ^4^Shanxi Agricultural UniversityTaigu, China

**Keywords:** rice, signaling peptide, CEP, grain size, cell size

## Abstract

Plant peptide hormones play an important role in regulating plant developmental programs *via* cell-to-cell communication in a non-cell autonomous manner. To characterize the biological relevance of C-TERMINALLY ENCODED PEPTIDE (CEP) genes in rice, we performed a genome-wide search against public databases using a bioinformatics approach and identified six additional CEP members. Expression analysis revealed a spatial-temporal pattern of *OsCEP6.1* gene in different tissues and at different developmental stages of panicle. Interestingly, the expression level of the *OsCEP6.1* was also significantly up-regulated by exogenous cytokinin. Application of a chemically synthesized 15-amino acid OsCEP6.1 peptide showed that OsCEP6.1 had a negative role in regulating root and seedling growth, which was further confirmed by transgenic lines. Furthermore, the constitutive expression of *OsCEP6.1* was sufficient to lead to panicle architecture and grain size variations. Scanning electron microscopy analysis revealed that the phenotypic variation of *OsCEP6.1* overexpression lines resulted from decreased cell size but not reduced cell number. Moreover, starch accumulation was not significantly affected. Taken together, these data suggest that the *OsCEP6.1* peptide might be involved in regulating the development of panicles and grains in rice.

## Introduction

Intercellular communication is a fundamental mechanism for coordinating the development of multicellular organisms. Plant peptide hormones have been recognized as an important signal mediating cell-to-cell communication in a non-cell autonomous manner and regulating many processes of biological relevance, including meristem maintenance, cell proliferation and root development (Katsir et al., 2011). Among the peptide families, the C-TERMINALLY ENCODED PEPTIDE (CEP) genes encode a mature post-translationally modified peptide of 15-amino acids, excised from a precursor including an N-terminal secretion signal (NSS), a variable domain, one or more CEP domains and a short C-terminal extension (Ohyama et al., 2008). To date, more than 900 *CEP* genes have been identified across plant genomes. *CEP* genes are predominantly present in gymnosperm and angiosperm plants, but absent from the earliest diverging lineages of plants, indicating that their emergence coincided with the evolution of seed plants. A preliminary phylogenetic analysis of *CEP* genes divided them into two groups based on the homology of CEP domains (Delay et al., 2013; Roberts et al., 2013; Ogilvie et al., 2014). Although *Arabidopsis CEP* genes are mainly expressed in vasculature, a spatiotemporal expression pattern is still observed for most members (Roberts et al., 2013). Recent emerging evidences have demonstrated that *CEP*s are involved in the regulation of plant root/shoot growth, lateral root and root nodule development (Ohyama et al., 2008; Delay et al., 2013; Imin et al., 2013; Roberts et al., 2013; Tabata et al., 2014; Mohd-Radzman et al., 2015). For example, *Arabidopsis* treated with chemically synthesized CEP1 peptide or ectopically expressing of *AtCEP1* exhibits repressed primary root growth and retarded lateral root elongation by reducing the cell number in the meristem zone and decreasing the cell size in the mature region (Ohyama et al., 2008). The ectopic expression of *MtCEP1* or exogenous application of the synthetic peptide reduces the number of emerged lateral roots; by contrast, simultaneous down-regulation of *MtCEP1, 2, 5*, and *11* dramatically increases lateral root numbers (Imin et al., 2013; Mohd-Radzman et al., 2015), whereas the *AtCEP*3 loss-of-function mutation promotes root development under high NaCl concentration and nitrogen-limiting condition (Delay et al., 2013). In addition, peptide application experiments or the constitutive expression of other six CEPs (*AtCEP2, AtCEP3, AtCEP4, AtCEP5, AtCEP6*, and *AtCEP9*) results in repressed root development (Ohyama et al., 2008; Delay et al., 2013; Roberts et al., 2013). However, the function of these peptides is significantly different in the control of above-ground traits, e.g., no significant changes are observed in *AtCEP1* overexpression lines, such as leaf number, flowering time and fruit set, whereas reduced leaf number and delayed flowering time are found in *AtCEP2* overexpression lines. Moreover, overexpression of *AtCEP3* shows leaf epinasty, leaf etiolation and small leaves (Ohyama et al., 2008; Delay et al., 2013; Roberts et al., 2013). Furthermore, CEP family peptides can be induced by local nitrate starvation and act as an ascending N-demand signal to the shoot to mediate up-regulation of nitrate transporter genes (Imin et al., 2013; Tabata et al., 2014; Mohd-Radzman et al., 2015). Peptide sequences always involve some post-translational modifications, including tyrosine sulfation, proline hydroxylation and hydroxyproline arabinosylation, which are known to modulate the ability and specificity of peptides for target receptor proteins by affecting peptide conformation (Matsubayashi, 2011). The synthetic MtCEP1 domain 1 peptides D1:HyP4,11 (hydroxylation occurred at Pro4 and Pro11) and D1:HyP7,11 increase the nodule number, whereas D1:HyP4,11 and D1:HyP4,7,11 inhibit the lateral root development (Mohd-Radzman et al., 2015). However, Delay et al. (2013) reported that both CEP9.1 and CEP9.1 H (hydroxylation occurred at Pro4 and Pro11) reduced the length of the primary root in *Arabidopsis*, although the CEP9.1 H activity was higher than CEP9.1 (Delay et al., 2013). In addition, Kondo et al. (2006) reported that proline hydroxylation of CLV3 showed no difference in root growth inhibition compared with the peptide without modifications (Kondo et al., 2006).

Rice grain yield is a complex agronomic trait that is multiplicatively governed by three main components: number of panicles per plant, number of grains per panicle and grain weight (Xing and Zhang, 2010). Previous studies have reported that the *MONOCULM1* mutant significantly decreased rice yield by negatively controlling tiller formation (Li et al., 2003). Whereas repression of the *GRAIN NUMBER1* (*Gn1a*), encoding cytokinin oxidase/dehydrogenase, promotes cytokinin accumulation in rice inflorescence meristem and increases grain number (Ashikari et al., 2005). A number of genes involved in the regulation of grain width and length are map-based cloned, including *GS3, GL3.1/qGL3, TGW6, GW2, qSW5/GW5, GS5*, and *GW8* (Fan et al., 2006; Song et al., 2007; Weng et al., 2008; Li et al., 2011; Qi et al., 2012; Wang et al., 2012; Ishimaru et al., 2013). Furthermore, the disruption of gene expression related to endosperm developmental processes leads to the failure of seed maturation and yield loss (Zhou et al., 2013). For example, the suppression of *MADS29* expression promotes programmed cell death (PCD) of the nucellus and the nucellar projection in rice, resulting in shrunken seeds (Nayar et al., 2013); overexpression of *OsFIE1* shortens the duration of the syncytial stage, causes precocious cellularization and ultimately leads to small seeds (Ishikawa et al., 2011; Folsom et al., 2014).

As a model system of monocots, 11 *CEP* genes have been identified in rice by a bioinformatics approach based on peptide sequence conservation (Delay et al., 2013; Roberts et al., 2013; Ogilvie et al., 2014). To date, no further investigation has been conducted to explore their biological importance. In this study, we performed a genome-wide search of *CEP* genes across the rice genome and identified another six *CEP* genes, overexpression of *OsCEP6.1* caused a series of phenotypic variations compared with the control, including reduced root length, decreased tiller number and repressed plant height. More interestingly, overexpression of *OsCEP6.1* resulted in small seeds *via* the reduction of cell size but not of cell number.

## Materials and methods

### Plant materials and growth conditions

Chinese rice cultivar Zhong Hua 17 (ZH17) was used for the experiments in this study. ZH17 and transgenic rice seeds were sterilized with diluted (1:3, v/v) NaClO for 15 min and rinsed with sterile water 5 times. Seed germination was placed in the dark at 28°C for 72 h. The 6-day-old seedlings were transferred to nutrient solution containing 1.425 mM NH_4_NO_3_, 0.323 mM NaH_2_PO_4_, 0.513 mM K_2_SO_4_, 0.998 mM CaCl_2_, 1.643 mM MgSO_4_, 0.168 mM Na_2_SiO_3_, 0.125 mM Fe-EDTA, 0.019 mM H_3_BO_3_, 0.009 mM MnCl_2_, 0.155 mM CuSO_4_, 0.152 mM ZnSO_4_, and 0.075 mM Na_2_MoO_4_, pH 5.5. The hydroponic experiments were conducted with a 16-h-light (30°C)/8-h-dark (22°C) photoperiod, and the solution was refreshed every 3 days (Wang et al., 2009; Dai et al., 2012). Fully filled grains were used to measure grain length and width of five overexpression lines (Li et al., 2004). Grain weight (calculated by 100 grains) was determined in triplicate, and converted to the 1000-grain weight (Wu et al., 2008; Mao et al., 2010). Statistically significant differences were detected using a two-sample *T*-test (SPSS ver 17.0, Chicago, USA) where appropriate.

### Genome-wide identification, chromosomal location, and phylogenetic analysis of rice *CEP* genes

Eleven rice *CEP*s have been predicted *via* homology BLAST against public databases (Delay et al., 2013; Ogilvie et al., 2014). Rice CEP domains and all *Arabidopsis* CEP domains were used as query sequences for protein BLAST against NCBI non-redundant reference proteins (http://blast.ncbi.nlm.nih.gov) and the Rice Genome Annotation Project (http://rice.plantbiology.msu.edu). Then, all the full-length CEP protein sequences were used to predict NSS by performing SignalP (http://www.cbs.dtu.dk/services/SignalP) and SecretomeP (http://www.cbs.dtu.dk/services/SecretomeP-2.0/) interface software. The position of *CEP* genes on the rice genome was identified by the Genome Browser (http://rice.plantbiology.msu.edu). Phylogenetic analyses of rice CEP domains were conducted using the neighbor-joining algorithm in MEGA5 (Tamura et al., 2011) considering 1000 replications with bootstrap analyses. Rice CEP domain conservation was shown by WebLogo plots (http://weblogo.berkeley.edu) (Crooks et al., 2004).

### Vector construction and rice transformation

For overexpression, the 321-bp CDS of *OsCEP6.1* was amplified using the primer pair of *OsCEP6.1-L* and *OsCEP6.1*-R, and the resulting PCR product was cloned into the pCAMBIA1300-Ubi vector. The plasmid was co-precipitated with golden particles and introduced into Zhonghua17 (ZH17). At the T2 generation, two independent transgenic lines were used for further analysis.

### RNA isolation and real-time quantitative PCR

The primary root and shoot of the 6-day-old seedlings; the leaf, palea, lemma and stamen at the rice heading stage; and four different length panicles were collected. The required tissues (primary root, shoot, leaf, palea, lemma, stamen, pistil, and panicles at four different developmental stages) were collected at the proper stages and frozen in liquid nitrogen. The total RNA was extracted using the Trizol RNA isolation protocol (Life Technologies, USA). The first strand of cDNA was synthesized from total RNA using the Superscript II RT Kit (Invitrogen, Carlsbad, CA, USA).

Real-time qPCR experiments were performed using SYBR Green PCR master mix (TaKaRa, Otsu, Japan) on CFX96 Real-Time PCR Detection System (Bio-Rad Laboratories, Inc., USA). Specific primer pairs for the qRT-PCR analysis were listed in Table S1. PCR conditions consisted of an initial step at 95°C for 3 min followed by 40 cycles of 95°C for 15 s, 59°C for 15 s, and 72°C for 30 s. Ct values were determined using the CFX96 software with default settings. Differences between the Ct values of target gene and *Actin1* were calculated as ΔCt = Ct_*targetgene*_–Ct_*Actin*1_, and the relative expression levels of target genes were determined as 2^−ΔCt^. The average values of 2^−ΔCt^ were used to determine the difference in gene expression. Data were analyzed as described above.

### Exogenous hormone treatment and synthetic CEP treatment

After the germination of rice seeds, the 6-day-old seedlings were transferred to nutrient solution with or without 10 μM NAA (1-naphthalene acetic acid), 1, 3, 5, 10, and 20 μM 6-BA (6-benzylaminopurine), 10 μM GA_3_ (gibberellic acid), 100 μM ABA (abscisic acid), 1 μM BR (Epibrassinolide), 100 μM MeJA (methyljasmonate) and 50 μM ACC (1-aminocyclo-propane-1-carboxylic acid) (Lee et al., 1996; Cheng et al., 2007; Kurakawa et al., 2007; Tanaka et al., 2009; Yang et al., 2011), 6 h after hormone treatment, the roots were collected. Total RNA was extracted and analyzed by real-time RT-PCR. Moreover, the uniformly germinated seeds were transferred into the nutrient solution with or without synthetic CEP peptides in the cylindrical glass bottle (diameter = 4 cm, height = 10 cm) for phenotypic detection, the solution was refreshed every 3 days. Synthetic CEP peptides (DSRPTAPGNSPGIGN) without modifications were synthesized by SBS Genetech (Beijing, China), and the purity of the synthetic peptides was greater than 95%, as assessed by reverse phase high-performance liquid chromatography (Figure S1). Data were analyzed as described above.

### Microscopic observations

The outer spikelet hull surfaces and the inner epidermal cells of the lemma were observed and imaged through scanning electron microscopy (TM-3000, Hitachi, Japan), The distance between the tubercles of outer surface (*n* = 200) and the cell length and width of inner epidermal cell (*n* = 250) were measured using ImageJ software (http://rsb.info.nih.gov/ij/). Data were analyzed as described above.

### Histological analysis

Spikelet hulls at heading stage and grains at 2, 5, and 8 days after pollination were fixed in FAA (50% ethanol, 5% glacial acetic acid and 5% formaldehyde) for 24 h, dehydrated in an ethanol series, and embedded in Paraplast (Sigma). Tissue sections (8 mm thick) were cut, mounted and stained with safranin T and fast green. Sections were photographed under the microscopy (Ti-U, Nikon, Japan). The cell length of 200 randomly selected cells were measured using ImageJ software (http://rsb.info.nih.gov/ij/). Data were analyzed as described above.

## Results

### Identification and characterization of novel CEP genes in rice

**Figure 1:**
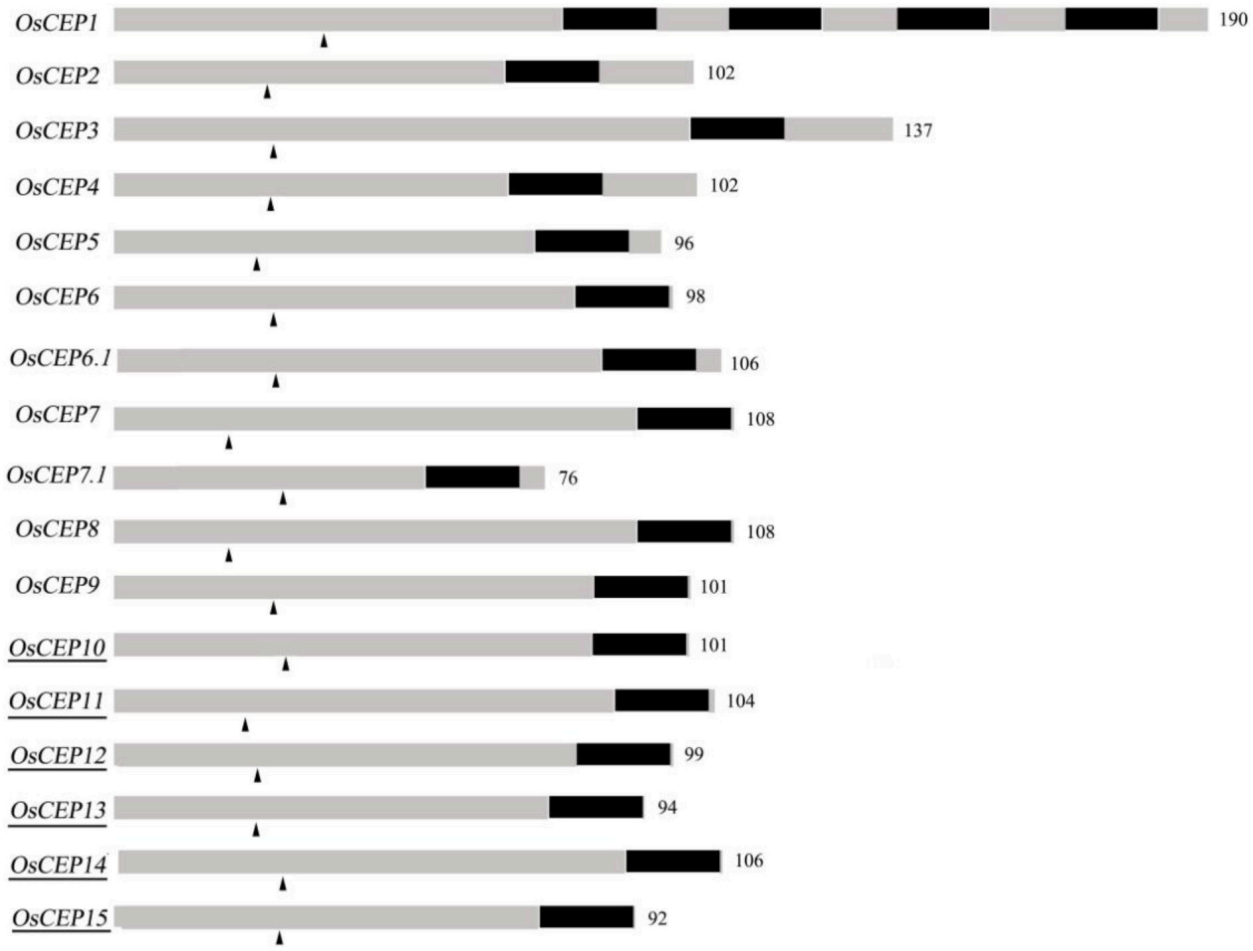
**Schematic representation of rice *CEP* genes**. Numbers are number of amino acids of the full length protein; Dark part, CEP domain; arrowhead, predicted signal peptide cleavage site; underscore, new identified *CEP*s.

**Figure 2:**
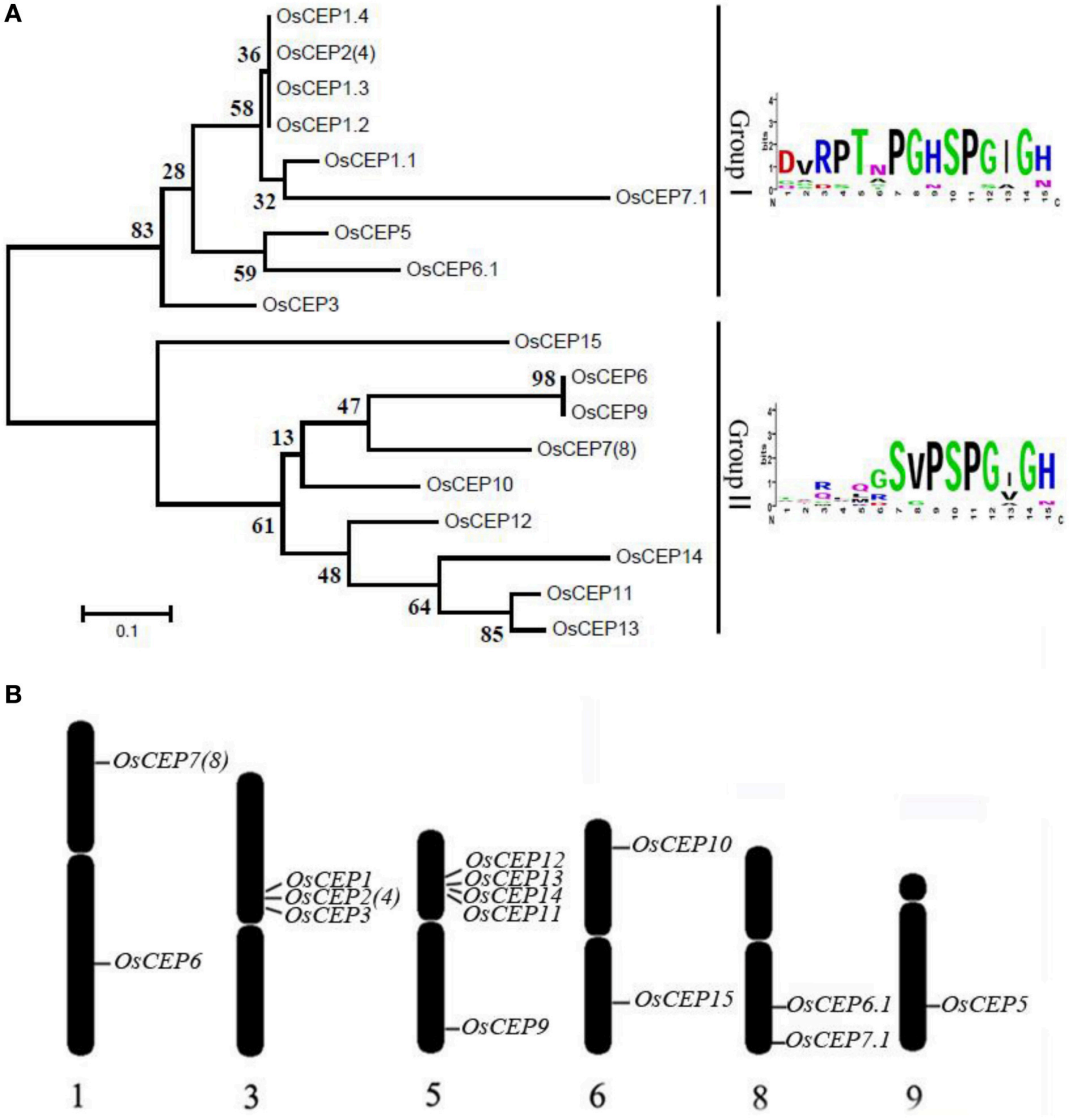
**Distribution of *CEP* genes found in the rice genome. (A)** Phylogenetic tree and WebLogo plot were used to show rice *CEP* gene clustering and the 15-amino acid CEP domain conservation. **(B)** Chromosomal distribution of *CEP* genes in the rice genome.

To identify new *CEP* genes in the rice genome, we searched the Rice Genome Annotation Project Database containing 49,066 gene models (http://rice.plantbiology.msu.edu/index.shtml) using all of the CEP domain sequences of *Arabidopsis* and rice, and six additional *CEP* genes were characterized (*OsCEP10*- *OsCEP15*) (Table S2), which were un-annotated before. Each of newly identified *CEP* genes encoded a small protein (92–106 amino acids) with a predicted signal peptide at the N-terminal and one CEP domain at the C-terminal (Figure 1). Phylogenetic analysis of 17 *CEP* genes showed that all of the newly identified *CEP*s belonged to group II (Figure 2A). Although having the same names, the sequences of *OsCEP6* and *OsCEP7* identified by Ogilvie et al. (2014) were different from those identified by Delay et al. (2013) (Figures S2A,B). Therefore, we renamed *OsCEP6* and *OsCEP7* identified by Ogilvie et al. (2014) as *OsCEP6.1* and *OsCEP7.1*, respectively. WebLogo plots showed that the CEP domain of group II was highly conserved among the nine amino acids at the C-terminal region except for the 8th amino acid and exhibited significant divergence in the first six amino acids at the N-terminal region, whereas the CEP domain of group I possessed highly conserved sequences of all 15-amino acids (Figure 2A). However, a divergence of the N-terminus sequences was found in both groups. Chromosome mapping showed that 17 *CEP*s were located on six out of ten rice chromosomes, and it is notable that *OsCEP1, OsCEP2* (*4*), *OsCEP3* and *OsCEP11, OsCEP12, OsCEP13, OsCEP14* were distributed in close proximity on chromosome 3 and 5, respectively (Figure 2B).

### *OsCEP6.1* exhibited a tissue-specific and hormone- induced expression pattern

**Figure 3:**
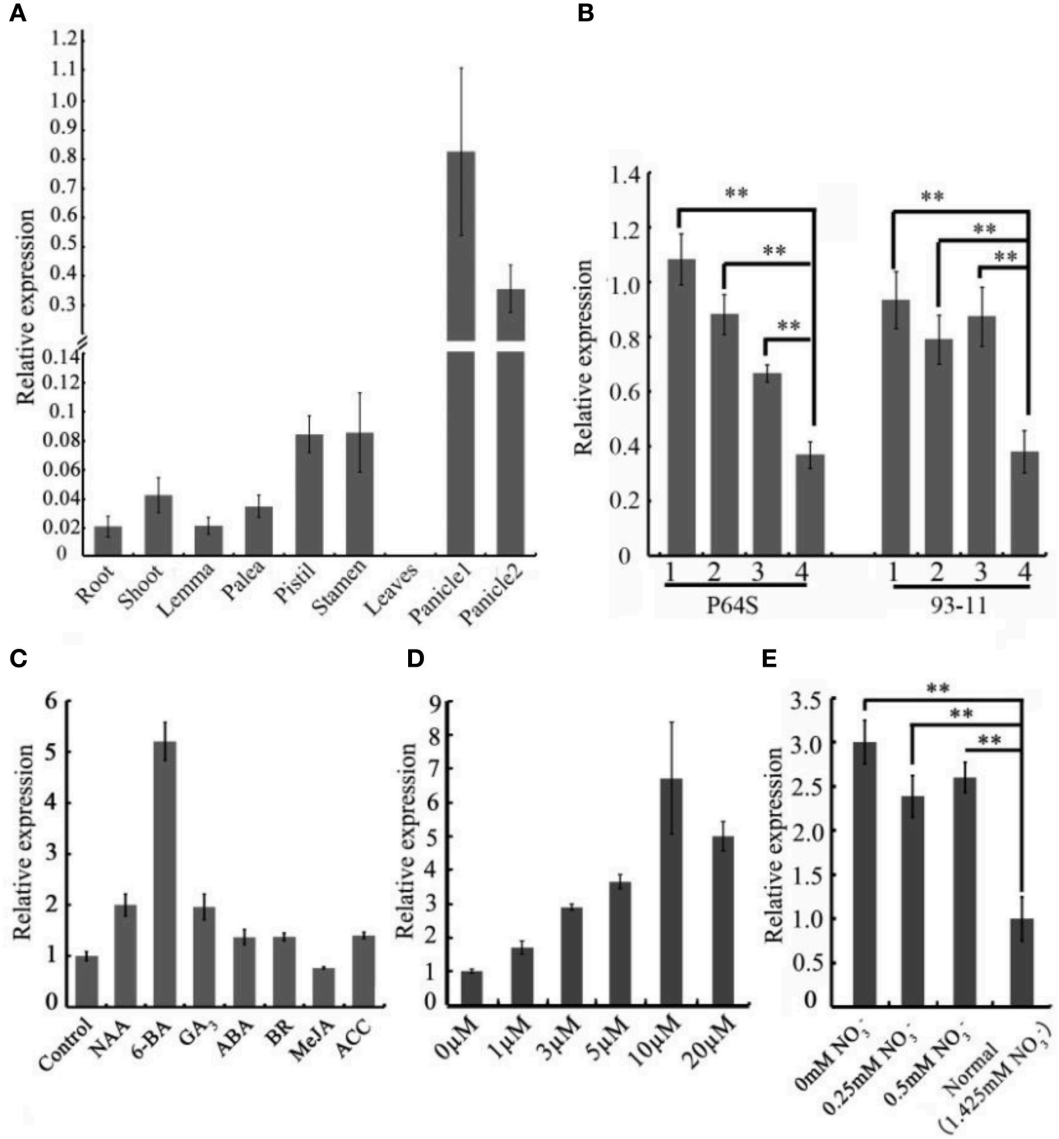
**The spatio-temporal expression patterns of *OsCEP6.1*. (A)** Quantitative RT-PCR analysis revealed that *OsCEP6.1* was highly expressed in the panicle among nine tissues of ZH17, including root, shoot, lemma, palea, stamen, pistil, leaf and panicles. panicle1: 40–50 mm panicles; panicle2: panicles at heading stage. **(B)** Quantitative RT-PCR analysis showed that the expression level of *OsCEP6.1* was significantly higher at the first three stages of panicles compared with the heading stage in both P64S and 93–11. 1: < 5 mm panicles; 2: 10–15 mm panicles; 3: 40–50 mm panicles, 4: panicles at heading stage. **(C)** The expression level of *OsCEP6.1* was significantly induced by 6-BA application. The 6-day-old seedlings were treated with different phytohormones (10 μM NAA, 10 μM 6-BA, 10 μM GA, 100 μM ABA, 1 μM BR, 100 μM MeJA, and 50 μM ACC). **(D)** The expression patterns of *OsCEP6.1* was significantly induced under 10 μM 6-BA compared with other concentration applications. The 6-day-old seedlings were treated with 0, 1, 3, 5, 10, and, 20 μM 6-BA. **(E)** The expression level of *OsCEP6.1* was increased under low nitrate concentration. The 6-day-old seedlings were transferred to the nutrient solutions with different nitrate concentration, and the root was collected 3 days after transfer. ^**^P < 0.01.

To gain an insight into the expression profiles of the *CEP* genes in rice, we searched the publicly available gene expression database on Rice qTeller (http://qteller.com/rice/). These *in silico* data indicated that *CEP* genes were expressed divergently and exhibited tissue-specific expression patterns. *OsCEP1, OsCEP2* (*4*), *OsCEP3, OsCEP12*, and *OsCEP13* were particularly expressed in root, whereas *OsCEP5* and *OsCEP6.1* were only expressed in early inflorescences and emerging inflorescences, respectively, displaying similar expression trends as previously reported (Ogilvie et al., 2014), and making these genes likely candidates for controlling rice reproductive development (Figure S3). To confirm the expression pattern of *OsCEP6.1*, we performed quantitative RT-PCR analysis in nine different tissues, and found *OsCEP6.1* was indeed highly expressed in reproductive tissues, particularly the panicle, which is consistent with Rice qTeller data (Figure 3A, Figure S3). To further investigate *OsCEP6.1* expression patterns, we examined its transcript abundance at four developmental stages of the panicle (< 5 mm, 10–15 mm, 40–50 mm and heading stage) in both the P64s and 93–11 varieties, showing significant down-regulation at heading stage compared with the first three stages (Figure 3B). Furthermore, we examined the expression pattern of *OsCEP6.1* in response to different plant hormones and found it was significantly induced by artificial 6-BA, especially at high concentrations, e.g., 10 and 20 μM, but not low concentrations, consistent with the previous reports (Imin et al., 2013), the significance of which needed further investigation (Figures 3C,D). In addition, nitrogen depletion also significantly induced the expression of *OsCEP6.1*, consistent with previous reports in *Medicago* and *Arabidopsis* (Delay et al., 2013; Imin et al., 2013; Tabata et al., 2014) (Figure 3E).

### Exogenous application of the synthetic peptide and ectopic expression of *OsCEP6.1* are sufficient to cause multiple phenotypic variations

**Figure 4:**
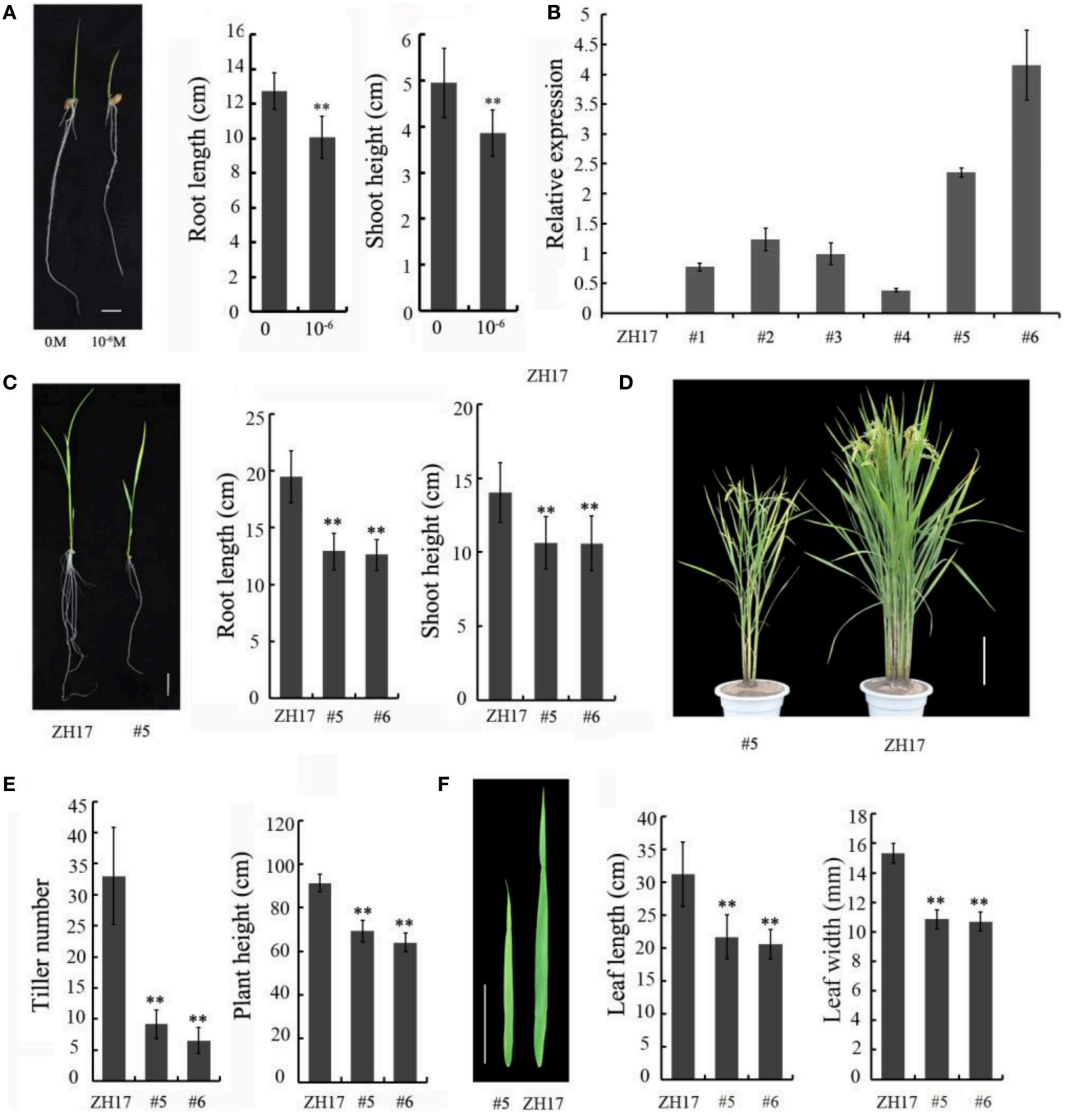
**Overexpression of *OsCEP6.1* negatively affected the vegetative growth of rice. (A)** Comparison of the root length and shoot height between the control and seedlings with synthetic CEP at the 8-day-old stage. The growth of shoot and root was significantly repressed by synthetic CEP peptide, by 17 and 20%, respectively. Bar = 1 cm. The values are means ± SD (*n* = 18), ^**^P < 0.01. **(B)** Quantitative RT-PCR analysis revealed the high expression level of *OsCEP6.1* in six overexpression lines at the T0 generation, particularly lines #5 and #6. **(C)** Comparison of the root length and shoot height of the transgenic lines (#5) and control at the 14-day-old seedlings. The growth of shoot and root was significantly repressed by approximately 25% in the transgenic line compared with the control. Bar = 2 cm. The values are means ± SD (*n*≥10), ^**^P < 0.01. **(D)** Overexpression of *OsCEP6.1* leads to phenotypic variation at the mature stage including plant stature and panicle architecture. Bar = 20 cm. **(E)** *OsCEP6.1* overexpression lines exhibited significantly fewer tillers and shorter height compared with those of the control. **(F)** Both the length and width of flag leaves were significantly smaller in the transgenic line (#5 and #6) than those in the control. Bar = 10 cm. The values are means ± SD (*n*≥12), ^**^P < 0.01.

**Figure 5:**
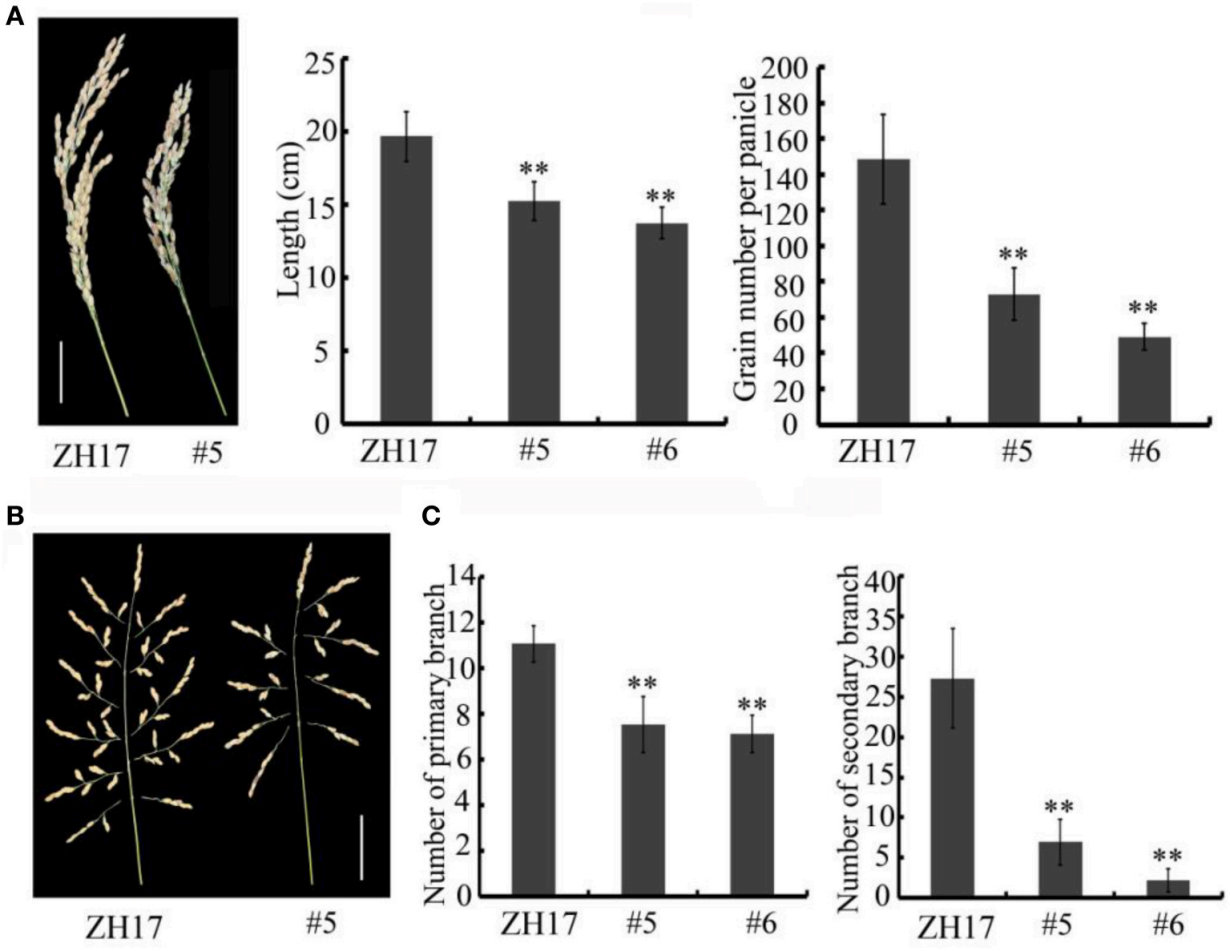
**Overexpression of *OsCEP6.1* modulates panicle architecture in rice. (A)** *OsCEP6.1* overexpression lines exhibited shorter panicles and fewer grains per panicle compared with the control (ZH17). Bar = 5 cm. The values are means ± SD (*n*≥12), ^**^P < 0.01. **(B)** Panicle branching. Bar = 5 cm. **(C)** The number of primary branches and secondary branches per panicle were significantly fewer in two transgenic lines (#5and #6) than in the control, the values are means ± SD (*n*≥12), ^**^P < 0.01.

**Figure 6:**
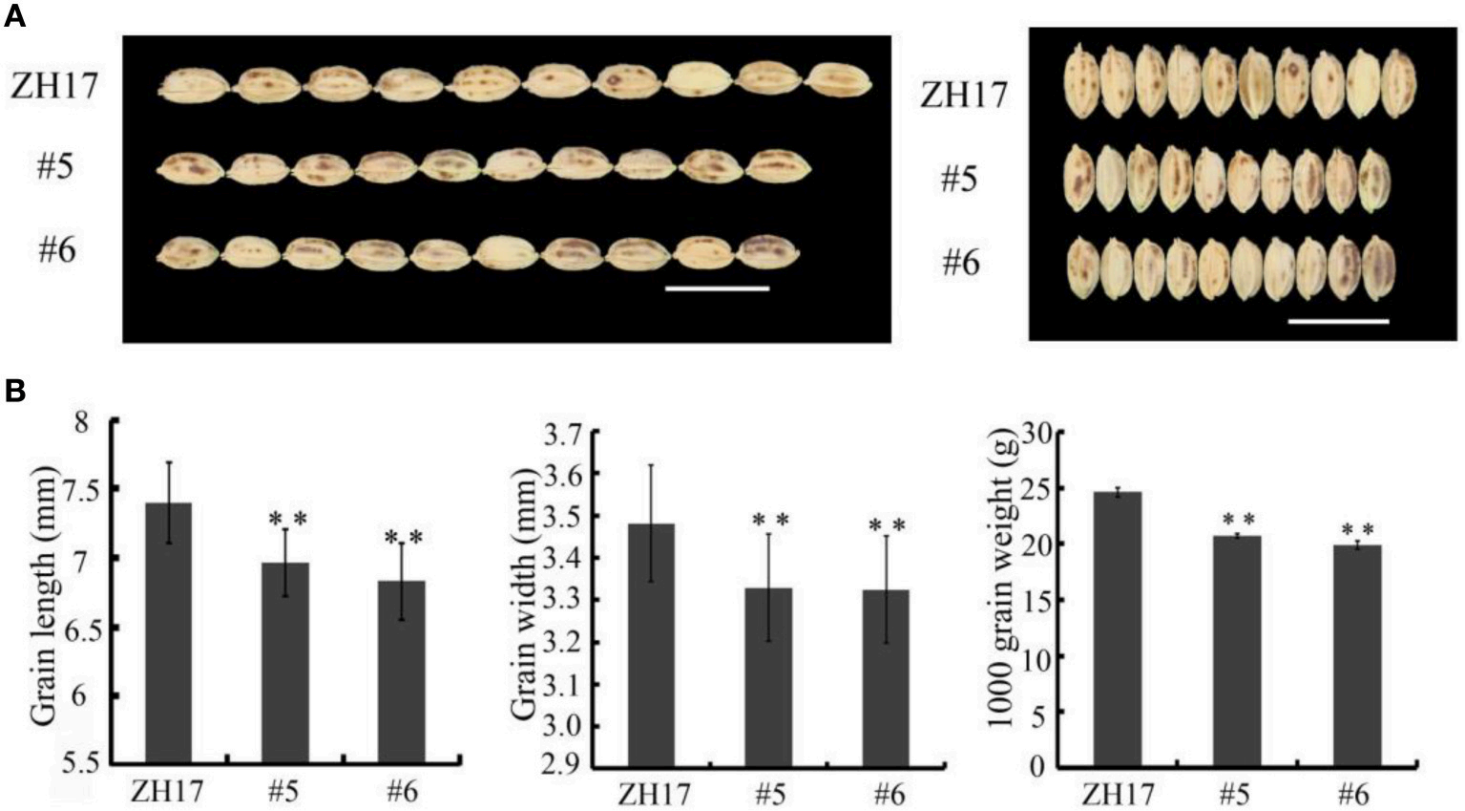
**Overexpression of *OsCEP6.1* decreases grain size in rice. (A)** Phenotypic variation between the control and *OsCEP6.1* transgenic line in both gain length and width. **(B)** Constitutive expression of *OsCEP6.1* significantly decreased seed length, seed width and 1000- grain weight, with 7, 4, and 19% reduction, respectively. Bar = 1 cm. The values are means ± SD (*n*≥90), ^**^P < 0.01.

To determine the biological relevance of *OsCEP6.1*, we first predicted and synthesized its putative 15-amino acid peptide (DSRPTAPGNSPGIGN) (Figure S1) and performed exogenous application experiment. Seedlings subjected to exposure of 10^−6^ M OsCEP6.1 peptide exhibited significantly reduced root and shoot development compared with the control. Specifically, synthesized peptide suppressed the shoot height and root length by approximately 17 and 20%, respectively (Figure 4A). Next, rice transgenic lines with constitutive expression of *OsCEP6.1* were generated under maize ubiquitin promoter. High *OsCEP6.1* expression levels were detected in leaves of the overexpression lines and two representative lines (#5 and #6) were selected for further analysis (Figure 4B). As expected, overexpression of *OsCEP6.1* fundamentally decreased the primary root length and seedling height by approximately 25% at the early stage in both transgenic lines, consistent with the phenotype caused by the exogenous simulation of synthetic peptide (Figure 4C). At the mature stage, the overexpression lines exhibited dramatically fewer tillers, shorter plant height and smaller flag leaves than those in the control plant ZH17 (Figures 4D–F). In addition, the panicle lengths of the two transgenic lines (#5and #6) were 22 and 30% shorter than those of ZH17, respectively (Figure 5A), and the number of primary and secondary rachis branches was also significantly fewer compared with the control, with 30 and 83% reduction, respectively (Figure 5B). Furthermore, constitutive expression of *OsCEP6.1* in rice negatively affected agronomic traits of the grain, including grain number per panicle, grain length, grain width and 1000 grain weight, which were severely reduction by 60, 7, 4, and 19%, respectively, in the transgenic lines (#5and #6) compared with those in ZH17 (Figures 5A, 6A,B).

### Overexpression of *OsCEP6.1* decreased cell size, not cell number in rice seeds

**Figure 7:**
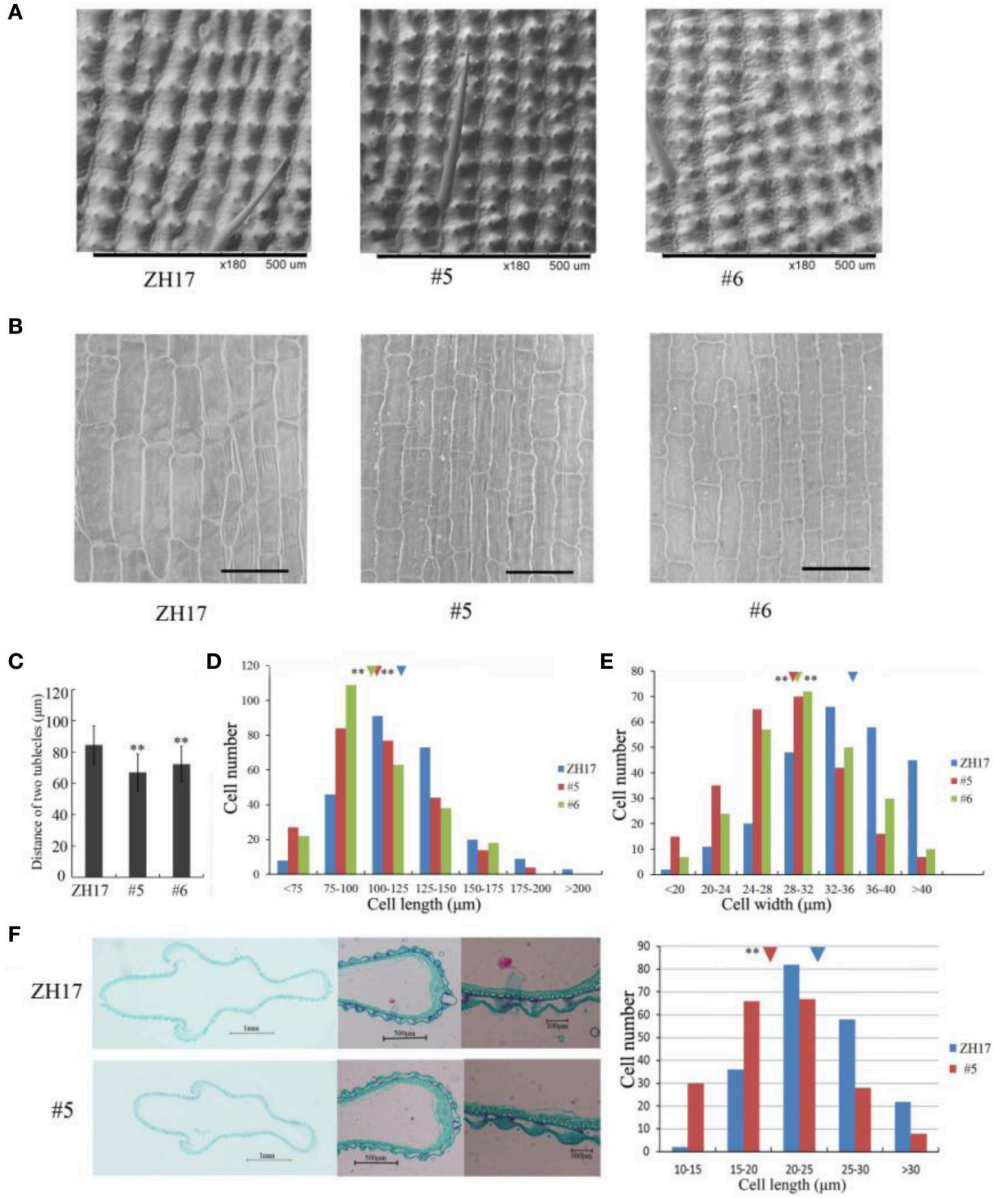
**Scanning electron microscopy analysis of *OsCEP6.1* overexpression lines and the control. (A)** Scanning electron microscopy analysis revealed that the distance between two adjacent tubercles on the outer spikelet hull surface was significantly shortened compared with the control (ZH17). Bar = 100 μm, × 180. **(B)** Scanning electron microscopy observation showed that the size of the lemma inner epidermal cell is smaller in the transgenic line than that in the control. Bar = 100 μm, × 250. **(C–E)** Statistical analysis of the distance between two adjacent tubercles (*n* = 200), cell length (*n* = 250) and cell width (*n* = 250) showed that all of them were significantly reduced by the overexpression of *OsCEP6.1*. The inverted triangles present the average cell length and width, the values are means ± SD. ^**^Indicates significance of cell length and width between the transgenic line and the control at the *P* = 0.01 level. **(F)** Histological analyses showed that the cell length of the outer parenchymal cell layers of the spikelet hulls is significantly larger in ZH17 than in the transgenic line. Blue and red inverted triangles show the average cell width of ZH17 and transgenic line (#5), respectively, and the values are means ± SD (*n* = 200), ^**^P < 0.01.

**Figure 8:**
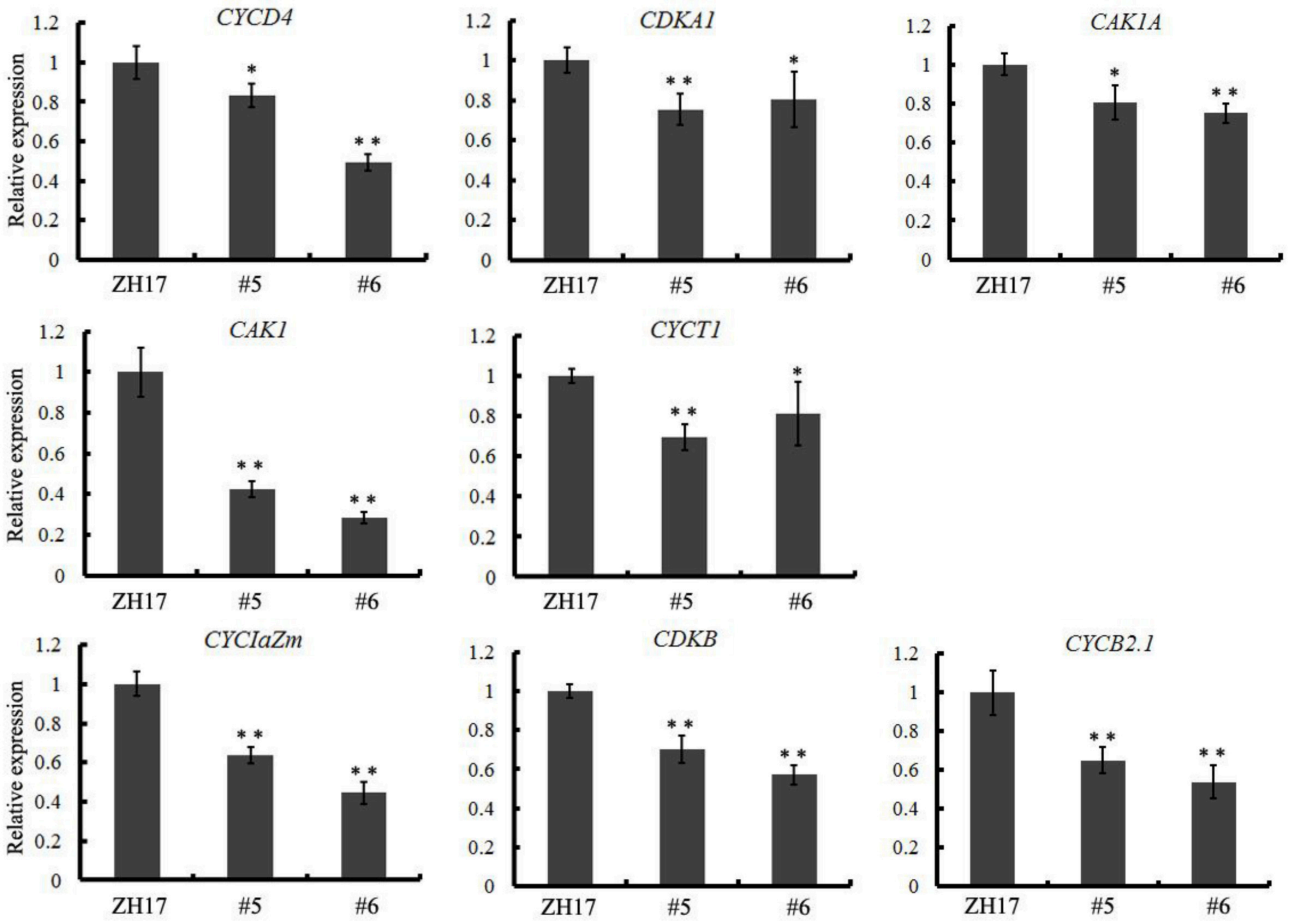
**Quantitative RT-PCR analysis revealed that eight cell-cycle-related genes, including five G1/S-phase genes (*CDKA1*, *CAK1*, *CAK1A*, *CYCT1*, and *CYCD4*) and three G2/M-phase genes (*CYCIaZm*, *CDKB*, and *CYCB2.1*), were significantly down-regulated in the transgenic line compared with the control**. The expression levels were determined using 6–8 cm young panicles from at least five plants, the values are means ± SD, ^*^P < 0.05, ^**^P < 0.01.

To investigate the causes underlying the phenotypic variation of grain size between transgenic and wild-type plants, we performed histological analysis to examine the stage transformation of endosperm in T2 transgenic lines, which was closely correlated with rice seed size (Folsom et al., 2014). Unexpectedly, the cellularizition of endosperm was faster in the control than in the transgenic lines at 2 days after pollination (Figure S4A), which was confirmed by the expression patterns of the syncytial stage specific MADS-box genes *OsMADS82* and *OsMADS87* (Figure S4B). However, the endosperm of ZH17 (control) was fundamentally larger than that of the *OsCEP6.1* overexpression lines at the respective developmental stages, which was inconsistent with the previous report that the precocious cellularization of endosperm leads to small seeds (Kang et al., 2008; Ishimaru et al., 2013; Folsom et al., 2014). Interestingly, the expression level of cellularization stage-specific *STRACH SYNTHASE II* (*SSII*) was significantly higher in transgenic lines than in ZH17 at early stages but lower at later stages (Figure S4B), motivating us to explore whether the starch was altered by constitutive expression of *OsCEP6.1*. Unfortunately, neither the starch granule nor the starch arrangements changed significantly in transgenic lines compared with wild type plants detected by scanning electron microscopy (Figure S5). Because the previous study reported that rice grain size is often greatly limited by the restriction of glume (Shomura et al., 2008), we next focused on the development of glume and compared the cell size between these two plants (Figure 7). Scanning electron microscopy observation revealed that the distance between two adjacent tubercles and the size of inner epidermal cells of the lemma (including cell length and width) were significantly smaller in the *OsCEP6.1* overexpression lines than in wild type plants (Figures 7A,B), and statistical analysis further confirmed our observation (Figures 7C–E). In addition, histological analysis of glume sectioning found that the cell length was reduced for most cells at outer parenchyma layer of the glume in the *OsCEP6.1* transgenic line than that in wild type plant (Figure 7F). However, no significant difference in the cell number was detected in the seeds between the transgenic lines and the wild type (Table S3), although the expression levels of five putative G1/S-phase genes (*CDKA1, CAK1, CAK1A, CYCT1*, and *CYCD4*), and three G2/M-phase genes (*CYCIaZm, CDKB*, and *CYCB2.1*) were down-regulated in the panicle of two transgenic lines (#5and #6) compared with the controls (Figure 8, Figure S6), suggesting that the changes of the cell-cycle related genes expression might be the secondary effects of *CEP6.1* overexpression but not the reason for seed size variation. Therefore, the above results collectively reveal that cell size, not cell number was significantly reduced because of constitutive expression of *OsCEP6.1* in rice, which resulted in the reduced grain size of rice in transgenic lines.

## Discussion

Previous reports have predicted more than 900 putative CEP peptides across the plant genome that predominantly occur in gymnosperm and angiosperm plants, but their biological significance remains largely unknown (Ohyama et al., 2008; Delay et al., 2013; Imin et al., 2013; Roberts et al., 2013; Ogilvie et al., 2014; Tabata et al., 2014; Mohd-Radzman et al., 2015). In this study, we performed a genome-wide survey of *CEP* genes in rice based on the CEP domain conservation and identified six additional *CEP* genes in rice, all belonging to Group II (Figure 2A). Of the previously identified eleven rice *CEP* members, the full length cDNA sequence of *OsCEP2* (EAY90324.1, 93–11) was likely the same as that of *OsCEP4* (AAP04189.1, Nipponbare), because they were mapped to the same position on the short arm of chromosome 3 with only one SNP between them, which was most likely caused by different subspecies (Figure 2B, Figure S7A). Similarly, *OsCEP7* (Os01g0203400.1) identified from Nipponbare was assumed to be *OsCEP8* (EAY72936.1) from 93 to 11 based on sequence similarity and consistent mapping position (Figure 2B, Figure S7B). In addition, we renamed *OsCEP6* and *OsCEP7*, which were identified by Ogilvie et al. (2014), as *OsCEP6.1* and *OsCEP7.1*, respectively, because of their sequence differences compared with previously identified *OsCEP6* and *OsCEP7* (Figure S2B). Thus, 15 *CEP* genes in total were identified in rice genome and exhibited a biased distribution on six chromosomes (Figure 2B). Specifically, *OsCEP11, 12, 13*, and *14* were tandemly arrayed in the middle of the short arm of chromosome 5, whereas *OsCEP1, 2(4)* and *3* were clustered proximally to the centromere of chromosome 3.

The importance of *CEP* genes in cell-to-cell communication in plants has been documented, which are involved in the regulation of plant growth and development (Ohyama et al., 2008; Delay et al., 2013; Roberts et al., 2013; Ogilvie et al., 2014; Mohd-Radzman et al., 2015), e.g., constitutive expression of *AtCEP3* inhibits primary root development in transgenic *Arabidopsis*, whereas the silencing of *AtCEP3* promotes root growth under the high salt concentration and nitrogen-limiting conditions (Delay et al., 2013). In *Medicago* ectopic expression of *MtCEP1* reduces the lateral root number, but the simultaneous down-regulation of *MtCEP1, 2, 5*, and *11* results in a contrasting phenotype. However, knockdown of *MtCEP1* alone does not show any significant phenotypic variation, which is probably due to the functional redundancy (Ishikawa et al., 2011; Imin et al., 2013; Mohd-Radzman et al., 2015). In this study, the tissue-specific expression pattern and the overexpression experiment indicated that *OsCEP6.1* might be involved in the regulation of panicle development, causing reduced plant height, tiller number, grain number and grain size, but the starch granule and the starch arrangement were not affected in the *OsCEP6.1* overexpression lines. Although *OsCEP6.1* was specifically expressed in rice panicles and ectopic expression of *OsCEP6.1* caused pleiotropic effects on plant reproductive growth, further efforts are still needed to verify its specific function on ear development. However, this is the first report regarding the *CEP* gene participating in the regulation of proliferative tissue development in rice. Therefore, we can conclude that *OsCEP6.1* is sufficient but not necessary to repress plant development based on our present data. *OsCEP5* and *OsCEP6.1* exhibit similar expression patterns among different tissues (Ogilvie et al., 2014), suggesting that they might have redundant functions, thus phenotypic variation of the panicle is expected in further analysis upon the knockdown of both *CEP*s.

The interesting phenotypic variation motivated us to further explore the underlying causes and found that reduced cell size but not cell number leads to small grain size, although eight cell-cycle-related genes were down-regulated in the *OsCEP6.1* overexpression lines (Figure 8). Further analysis found that five out of 17 grain-size-related genes (*GW2, GW5, GS3, GL3.1*, and *SG1*) were significantly down-regulated in *OsCEP6.1* constitutive expression lines (Figure S8), through which *OsCEP6.1* might contribute to the delayed development of rice grain. Previous studies have suggested that precocious cellularization of endosperm will always result in small endosperm because of few free nuclei (Ishikawa et al., 2011; Zhou et al., 2013; Folsom et al., 2014), but our histological analysis exhibited opposite situation in that overexpression of *OsCEP6.1* caused the slower cellularizaiton of endosperm and smaller seeds in transgenic line compared with the control. This phenotypic variation maybe be involved in the down-regulation of cell-cycle-related genes, which results in delayed growth at all developmental stages. Together, this study identified six new *CEP* genes in the rice genome and verified that overexpression of *OsCEP6.1* can result inpleiotropic effects on growth decreasing in rice, which sheds light on the understanding of the potential role of *CEP* genes during rice development.

## Author contributions

ZN, MX conceived the project. ZS, TW, GX, and HL collected the plant materials, ZS and TW performed the experiment, YL, MZ, and RX analyzed data. MX and NZ wrote the manuscript.

### Conflict of interest statement

The authors declare that the research was conducted in the absence of any commercial or financial relationships that could be construed as a potential conflict of interest.

## Supplementary material

Figure S1 **MS data of synthetic CEP peptides (DSRPTAPGNSPGIGN)**.

Figure S2 **Nucleotide sequences alignment of the rice *CEP* genes. (A)** The sequences of *OsCEP1, 2, 3*, and *5* identified in Ogilvie's paper (red) are the same as those identified by Delay et al. (2013) and Roberts et al. (2013) (black). **(B)** The sequences of *OsCEP6* and *7* identified in Ogilvie's paper (red) are different from those of *OsCEP6* and *7* identified by Delay et al. (2013) and Roberts et al. (2013) (black).

Figure S3 **Expression patterns of 14 rice *CEP*s as determined by Rice qTeller (http://qteller.com/rice/)**.

Figure S4 **Histological analysis of the rice grain and expression profile of syncytial and cellularization stage specific genes. (A)** The *OsCEP6.1* overexpression line exhibited slower cellularization than that of the control. **(B)** The expression level of syncytial stage specific MADS-box genes (*OsMADS82*and *OsMADS87*, but not *AGL36*) was significantly higher in the transgenic line than in the control at 2 DAP, whereas the expression level of the cellularization stage specific gene *STRACH SYNTHASE II* (*SSII*) was higher at 5 DAP but lower at 8 DAP in the transgenic line compared with the control.

Figure S5 **Scanning electron microscopy analysis revealed that neither the starch granule nor the starch arrangement was obviously different between the control (ZH17) and the transgenic lines (#5)**.

Figure S6 **Quantitative RT-PCR analysis revealed that eight cell-cycle-related genes, including five G1/S-phase genes (*CDKA1, CAK1, CAK1A, CYCT1*, and *CYCD4*) and three G2/M-phase genes (*CYCIaZm, CDKB* and *CYCB2.1*), were significantly down-regulated in the transgenic line compared with the control**. The expression levels were determined using 6–8 cm young panicles from at least five plants, the values are means ± SD, ^*^*P* < 0.05, ^**^*P* < 0.01.The inner control genes was the *UBQ* gene.

Figure S7 **(A)** *OsCEP2* (EAY90324.1, 93–11) and *OsCEP4* (AAP04189.1, Nipponbare) have only one SNP in their nucleotide sequences. **(B)** *OsCEP7* (Os01g0203400.1, Nipponbare) and *OsCEP8* (EAY72936.1, 93–11) have 98% similarity between each other.

Figure S8 **Quantitative RT-PCR analysis revealed that five grain size related genes (*GL3.1, SG1, GW2, GW5*, and *GS3*) were down-regulated in the two transgenic lines (#5and #6) compared with the control**. Expression levels were determined using 6–8 cm young panicles from at least five plants, the values are means ± SD, ^*^*P* < 0.05, ^**^*P* < 0.01.

Table S1 **Primer pairs used in this study**.

Table S2 **The rice *CEP* genes identified in the previous study (Delay et al., 2013; Roberts et al., 2013; Ogilvie et al., 2014) and our study**.

Table S3 **Cell size and cell number in the seeds**.

## Abbreviations

ZH17: Zhong Hua 17
*CEP*: *C-TERMINALLY ENCODED PEPTIDE*
DAP: days after pollination
*SSII*: *STRACH SYNTHASE II*.
