# Supplementary material for: Overexpression of Peptide-Encoding OsCEP6.1 Results in Pleiotropic Effects on Growth in Rice (O. sativa)
Source: Front Plant Sci. 2016 Mar 2;7:228. doi: 10.3389/fpls.2016.00228 (PMC4773640; doi:10.3389/fpls.2016.00228)
Supplement: Figure S1 — MS data of synthetic CEP peptides (DSRPTAPGNSPGIGN). [file DataSheet1.docx]

**Table S1** Primer pairs used in this study.

| gene | sequence |
| --- | --- |
| *OsCEP6.1-F* | CGGGGTACCATGGCGGCCAGTTCCAAGGT |
| *OsCEP6.1-R* | GGACTAGTCTAGTTGTTGATCTTCGCTT |
| *RTOsCEP6.1-F* | TACAAGGAAGCATGCCAATG |
| *RTOsCEP6.1-R* | TCTTCCCCTTGTTGCCTATG |
| *Actin1-L* | TGCTATGTACGTCGCCATCCAG |
| *Actin1-R* | AATGAGTAACCACGCTCCGTCA |
| *CYCD3-F* | CCTTCCACACTGACGGTACAGTT |
| *CYCD3-R* | TGCCGCTGCCAAATAGACA |
| *CYCD4-F* | GCCATGGAGTTGATACATCCAA |
| *CYCD4-R* | CCAGTAGGGCTCCGTGGAAT |
| *CAK1-F* | GACGGTCAGATTAGACGCAAGA |
| *CAK1-R* | TCCAAAGGATGTCCACA |
| *CAK1A-F* | GACCGACAAGGGTTTCAGCAT |
| *CAK1A-R* | CCAGCATGTTCAGGAAGATACAAT |
| *CDKA1-F* | GGTTTGGACCTTCTCTCTAAAATGC |
| *CDKA1-R* | AGAGCCTGTCTAGCTGTGATCCTT |
| *CDKA2-F* | CGAGATTTGAAGCCCCAGAA |
| *CDKA2-R* | TCCGCGAGCTTCAATGAGTT |
| *CYCT1-F* | GCATTTGTTGCAGCTCAAG |
| *CYCT1-R* | TCACCACTTCGCTGACTTATTG |
| *E2F2-F* | TGTTGGTGGCTGCCGATAT |
| *E2F2-R* | CGCCAGGTGCACCCTTT |
| *H1-F* | GCAAGGCACCTGCAGCTT |
| *H1-R* | AGGCAGCCTTTGTACAGATCCT |
| *MCM2-F* | AAGTTGGCAAAAGATCCACGG |
| *MCM2-R* | CCCCCAAACATAGCTAGTGCAA |
| *MCM3-F* | TTCATGCGTCACTAAATGCGAG |
| *MCM3-R* | TGAATCTGGAAGCCCAATGTTC |
| *MCM4-F* | CCCGAATGCGATTCTCTGAA |
| *MCM4-R* | ACCAGTGGCATGATCAGTTGC |
| *MCM5-F* | AAGGAGAACTGCCTGTCCATGA |
| *MCM5-R* | AGTGGCCTTAGCTTTCACCCTC |
| *CDT2-F* | AACCGCACCAAACACTGGAA |
| *CDT2-R* | GCAATTCACCATCTGCACTGG |
| *CYCA2.1-F* | AGGTTGTCAAGATGGAGAGCGA |
| *CYCA2.1-R* | CGCTTTTTGTCTTCCTGGCA |
| *CYCA2.2-F* | AGGTTGTCAAGATGGAGAGCGA |
| *CYCA2.2-R* | CGCTTTTTGTCTTCCTGGCA |
| *CYCA2.3-F* | GTTTCGGTTGACGAGACGATGT |
| *CYCA2.3-R* | CGCTGCAAGGAACCTAGAACTG |
| *CYCB2.1-F* | AAGTTTGGCCAGGAGTGAGCA |
| *CYCB2.1-R* | TCAAGAGCATCAGCGTCGAGA |
| *CYCB2.2-F* | CTCAAGGCTGCACAATCTGACA |
| *CYCB2.2-R* | GCATTGACGGCTGGAATTTG |
| *CYCIaZm-F* | CACTCTCAAGCACCACACTGGA |
| *CYCIaZm-R* | ACAACCCTCAGCTTGCTCTCAG |
| *CDKB-F* | AAGTTTGGCCAGGAGTGAGCA |
| *CDKB-R* | TCAAGAGCATCAGCGTCGAGA |
| *MAPK-F* | ACAGAGCAGCCGAATTTTGAGA |
| *MAPK-R* | TTCAGCGAAGCTCACACTTGG |
| *KN-F* | CACCAGCTTCAAGAGATCGTGA |
| *KN-R* | CCGGAATTGAGACACAACTGC |
| *CDC20-F* | TCGAATCACCTGTTTGTTGGC |
| *CDC20-R* | TGGAGACAATCCAACGCAAAG |
| *MAD2-F* | GAGCCATGCATATTCGACGTG |
| *MAD2-R* | GGTGTCGAAGGAATGCAGCTT |
| *PGL2-L* | ATGTCGAGCAGAAGGTCGTC |
| *PGL2-R* | TCAGGAGCGGAGGATGCTGC |
| *PGL1-F* | GCTAAGTGGGGGTGTCAAGA |
| *PGL1-R* | GTCTTGTGGCGCCTGATAAT |
| *GS3-F* | CATCGGAGAAGCGAAGTCAT |
| *GS3-R* | TTGAGGTTGAAGGAGGAGGA |
| *BRD1-F* | ACCTCGATGATGGCTGTCAA |
| *BRD1-R* | AACAGCTCGAGTGAAGGTCA |
| *GL3.1-F* | TCACAACTCCCAGGATAGG |
| *GL3.1-R* | TTTGTCTCGCTCGCTCAT |
| *GIF1-F* | CATCGCGCAACCCGAACATG |
| *GIF1-R* | TGTCGATCAGGCTCCTCAGAG |
| *FLO2-F* | CACACCCTCCAGCAATATCA |
| *FLO2-R* | CCTTCTGCGACTGCTTTTCT |
| *SRS3-F* | GCAACAGCAACAACGGCCGAC |
| *SRS3-R* | TGTGTGCTGCTATGAGAGCCTCC |
| *GS5-F* | CATTCCATGCAAATGCCAGTGGAC |
| *GS5-R* | CAGCCCTGCTTTGATGAGCTTG |
| *GW5-F* | AGGTGGTGGTGGTGGAGTCC |
| *GW5-R* | GCGTGGCGGTCGTTCTCG |
| *HGW-F* | ACAACTCCCACTACTTCTGTGGCT |
| *HGW-R*  | *TTGAGGATGTGAAGCCCATCTCGT* |
| *GW8-F* | AGGAGTTTGATGAGGCCAAG |
| *GW8-R* | GCGTGTAGTATGGGCTCTCC |
| *SG1-F* | CCCAGATGGATCTTGGAGGT |
| *SG1-R* | GGCAGACAGCAAGCTGAAAG |
| *SRS5-F* | ATGAGGGAGTGCATCTCGAT |
| *SRS5-R* | CAAGATCGACGAAGACAGCA |
| *UBQ-L* | AACCAGCTGAGGCCCAAGA |
| *UBQ-R* | ACGATTGATTTAACCAGTCCATGA |

**Table S2**. The rice *CEP* genes identified in previous study ([Delay et al., 2013](#_ENREF_5);[Roberts et al., 2013](#_ENREF_26)) and our study.

| **MSU ID** | **RAP locus ID** | **Gene name** | **ORF length(bp)** | **chromosome Lucas** | **SignalP** | **CEP domian name** | **CEP domian sequence** |
| --- | --- | --- | --- | --- | --- | --- | --- |
| LOC_Os03g27680 | no hits | *OsCEP1* | 573 | Chr3:15867188-15863777 | 0.997 | OsCEP1.1 | GVRPTNPGHSPGIGH |
|  |  |  |  |  |  | OsCEP1.2 | DVRPTNPGHSPGIGH |
|  |  |  |  |  |  | OsCEP1.3 | DVRPTNPGHSPGIGH |
|  |  |  |  |  |  | OsCEP1.4 | DVRPTNPGHSPGIGH |
| LOC_Os03g27690 | no hits | *OsCEP2* | 309 | Chr3:15873673 - 15873981 | 0.992 | OsCEP2 | DVRPTNPGHSPGIGH |
| LOC_Os03g27740 | no hits | *OsCEP3* | 414 | Chr3:15901680 - 15902093 | 0.809 | OsCEP3 | DARPTAPGHSPGAGH |
| LOC_Os03g27690 | no hits | *OsCEP4* | 309 | Chr3:15873673 - 15873981 | 0.997 | OsCEP4 | DVRPTNPGHSPGIGH |
| LOC_Os09g28780 | no hits | *OsCEP5* | 291 | Chr9:17481453 - 17481743 | 0.937 | OsCEP5 | DGRPTSPGHSPGIGN |
| LOC_Os01g53010 | Os01g0730400 | *OsCEP6* | 297 | Chr1:30468116 - 30467820 | 0.878 | OsCEP6 | TERLMRSVPSPGVGH |
| LOC_Os08g37070 | no hits | *OsCEP6.1* | 321 | Chr8:23425536 - 23425856 | 0.968 | OsCEP6.1 | DSRPTAPGNSPGIGN |
| LOC_Os01g10640 | Os01g0203400 | *OsCEP7* | 327 | Chr1:5685594 –  5686401 | 0.994 | OsCEP7 | SSRMLGSVPSPGVGH |
| no hits | Os06g0710001 | *OsCEP7.1* | 232 | Chr6:30033491 - 30033721 | 0.721 | OsCEP7.1 | QVDSTTPGHSPSIGH |
| LOC_Os01g10640 | Os01g0203400 | *OsCEP8* | 327 | Chr1:5685594 – 5686401 | 0.994 | OsCEP8 | SSRMLGSVPSPGVGH |
| LOC_Os05g45380 | no hits | *OsCEP9* | 306 | Chr5:26328619 - 26328924 | 0.969 | OsCEP9 | TERLMRSVPSPGVGH |
| LOC_Os06g05960 | no hits | *OsCEP10* | 306 | Chr6:2732800 –  2733105 | 0.982 | OsCEP10 | AARFLGSVPSPGIGH |
| LOC_Os05g11620 | no hits | *OsCEP11* | 315 | Chr5:6582133 –  6582888 | 0.973 | OsCEP11 | AIQVQGSVPSPGIGH |
| LOC_Os05g11580 | no hits | *OsCEP12* | 300 | Chr5:6559647 –  6560352 | 0.994 | OsCEP12 | GWMPQGSVPSPGIGH |
| LOC_Os05g11600 | no hits | *OsCEP13* | 285 | Chr5:6571105 –  6570821 | 0.981 | OsCEP13 | KIQVQGSVPSPGIGH |
| LOC_Os05g11610 | Os05g0206000 | *OsCEP14* | 321 | Chr5:6576138 –  6576715 | 0.962 | OsCEP14 | TAQLQGSVPSPGIGN |
| LOC_Os06g43080 | Os06g0637400 | *OsCEP15* | 279 | Chr6:25895947 - 25896426 | 0.961 | OsCEP15 | LGGIKDSGPSPGAGH |

The CEP genes identified in previous study (Delay et al., 2013; Roberts et al., 2013; Ogilvie et al., 2014) are indicated with underscores.

**Table S3.** Cell size and cell number in the seeds.

|  | Length | Width |
| --- | --- | --- |
| Seed (mm) |  |  |
| ZH17 | 7.40±0.29 | 3.47±0.14 |
| #5 | 6.97±0.24** | 3.33±0.13** |
| #6 | 6.79±0.28** | 3.26±0.18** |
| Cell (μm) |  |  |
| ZH17 | 120.83±26.79 | 34.21±6.18 |
| #5 | 107.27±26.02** | 30.44±5.67** |
| #6 | 104.37±26.22** | 30.79±5.09** |
| Deduced cell number |  |  |
| ZH17 | 61.24 | 101.43 |
| #5 | 64.98 | 109.41 |
| #6 | 65.06 | 105.88 |

Average length and width of seeds are calculated using ≥ 90 seeds. Average cell length and width are calculated using 250 cells (mean ± SD). ** P < 0.01; The deduced cell number was calculated by dividing the seed length by the cell length based on Kitagawa et al. (2010).

**Figure S1**

**
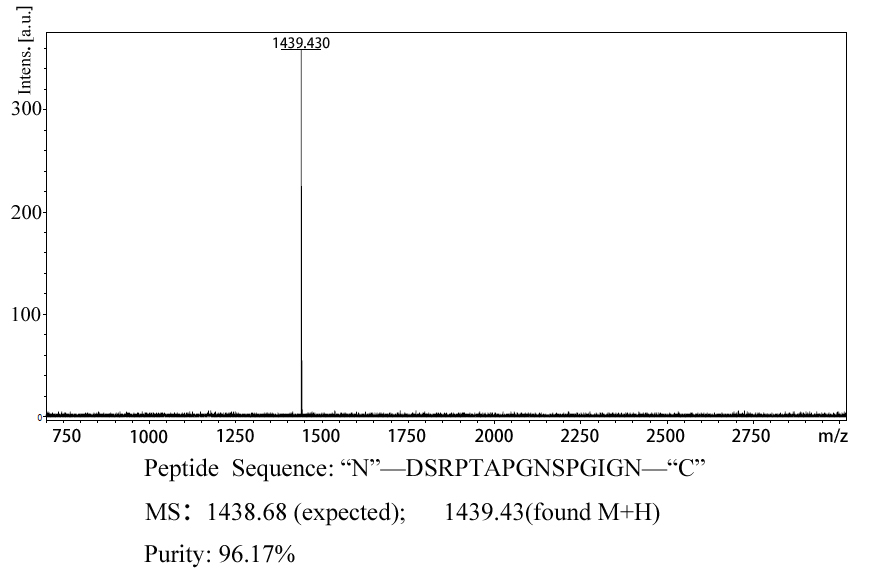
**

**Figure S2**

**
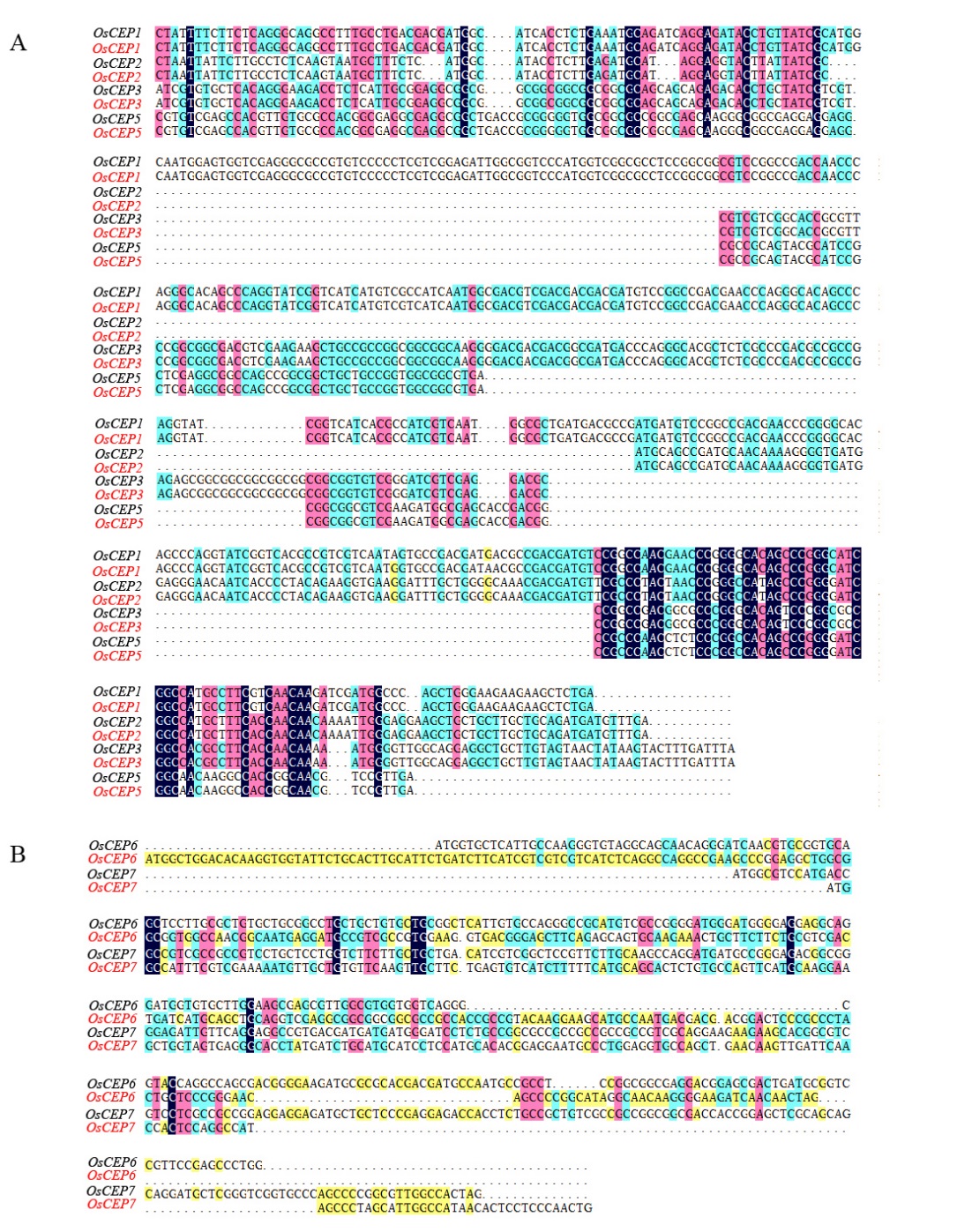
**

**Figure S3**

**
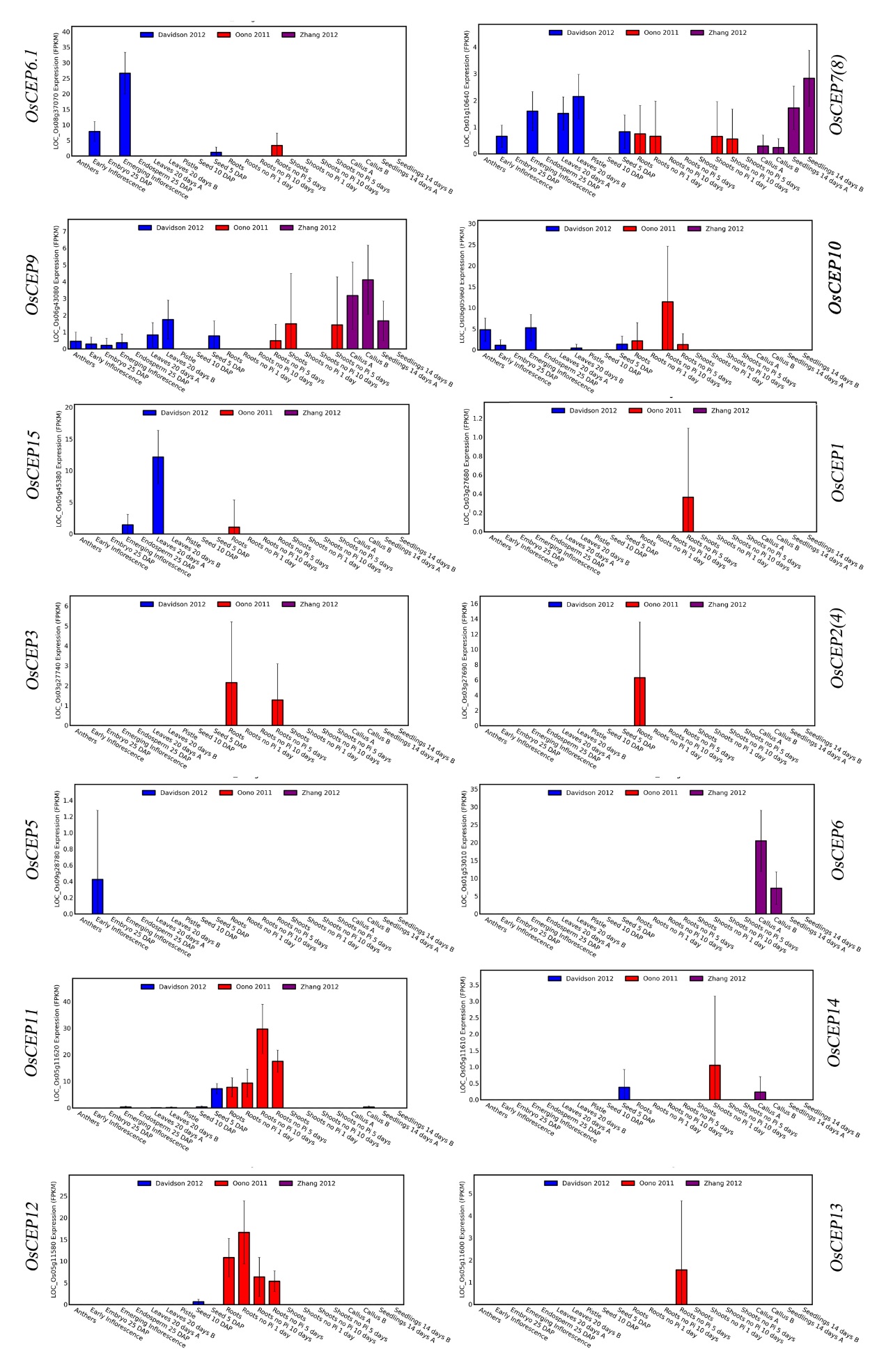
**

**Figure S4**

**
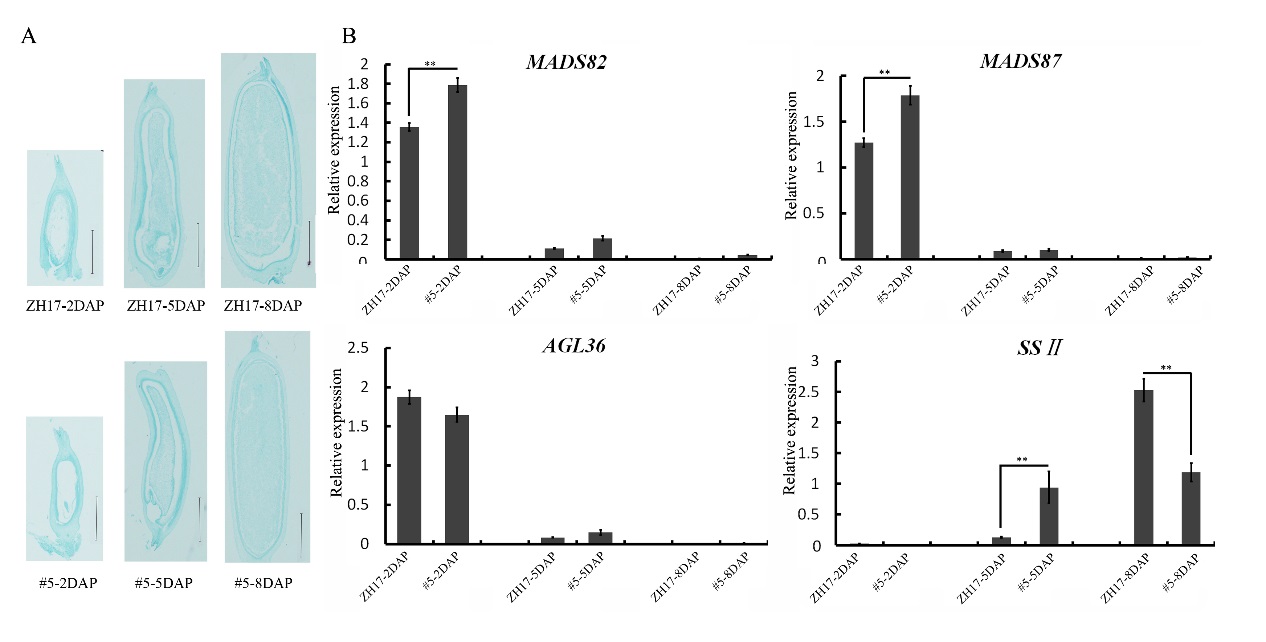
**

**Figure S5**

**
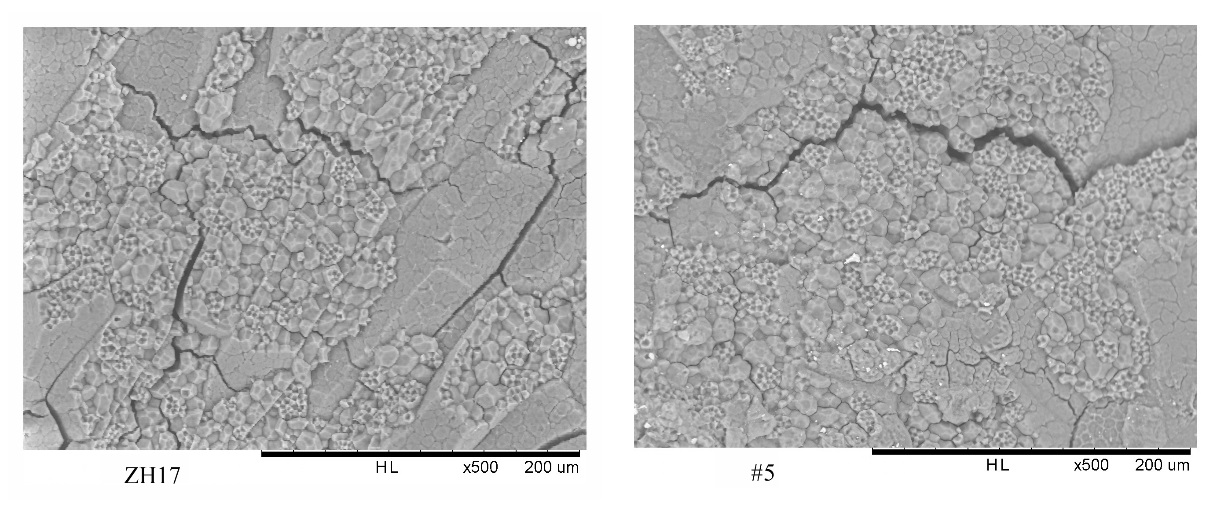
**

**Figure S6**

**
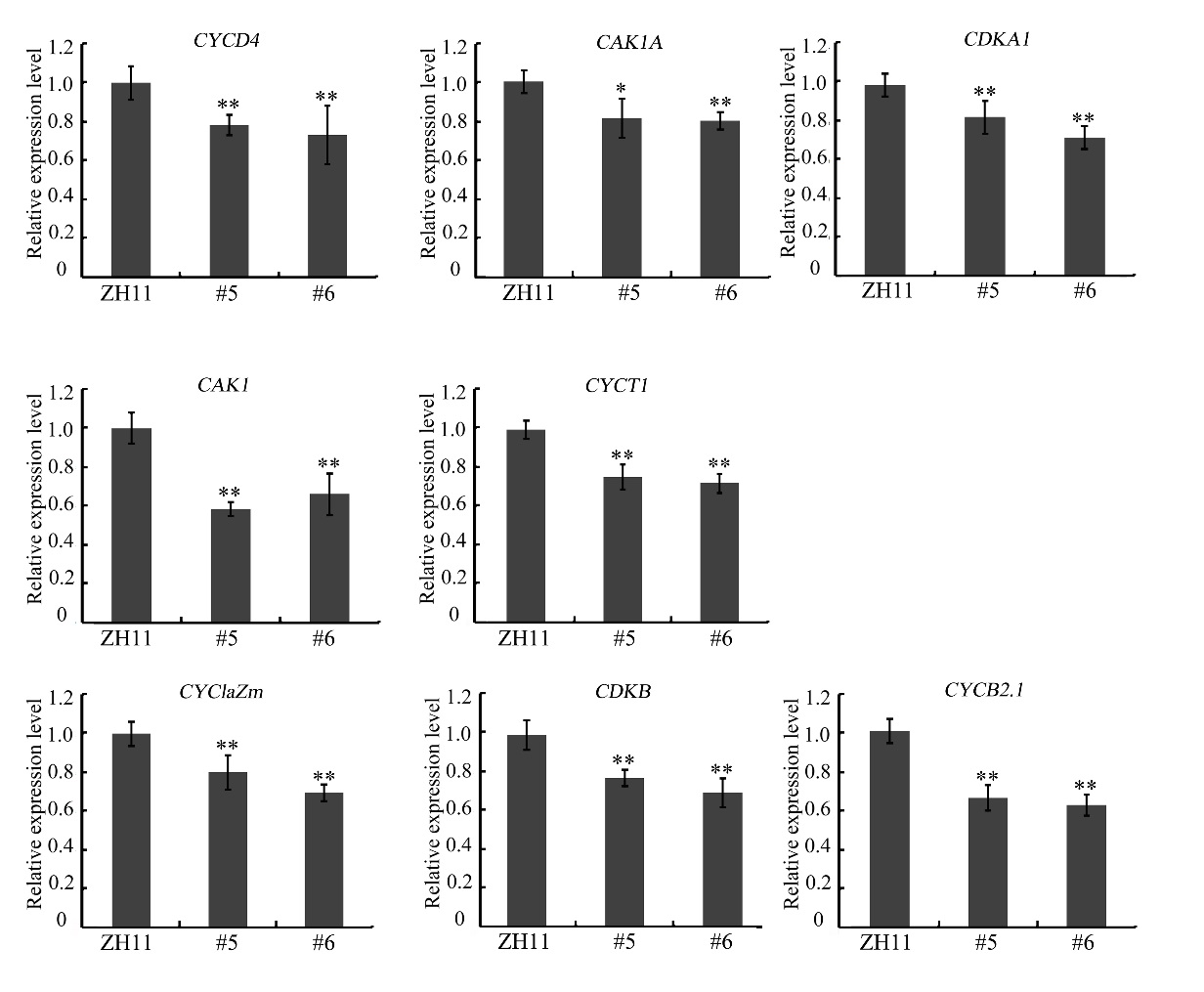
**

**Figure S7**

**
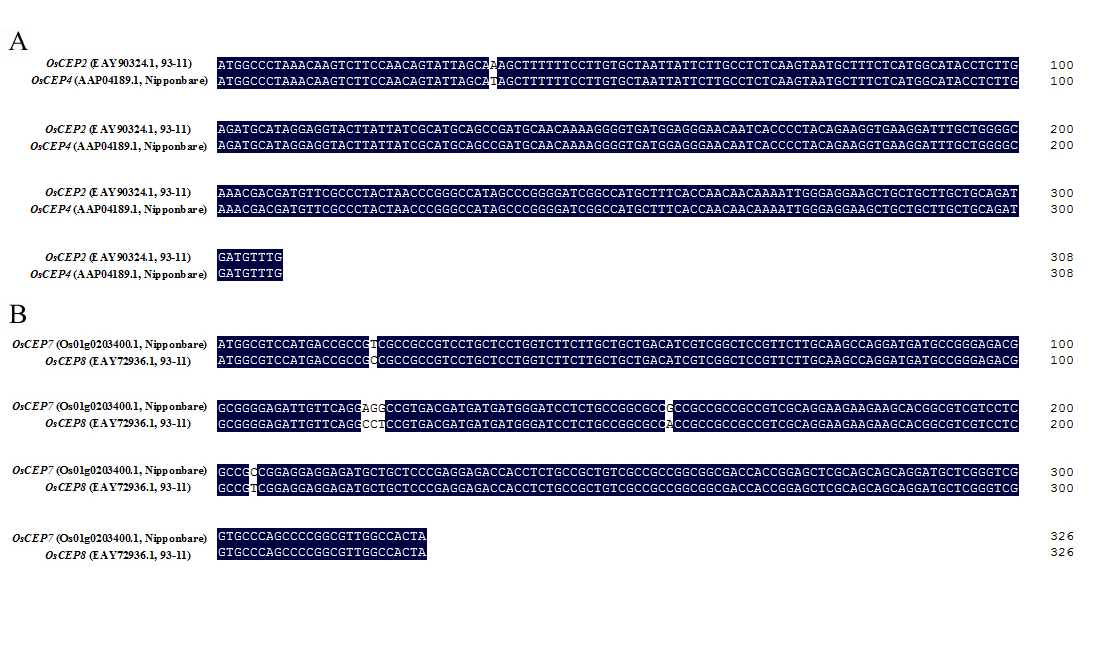
**

**Figure S8**

**
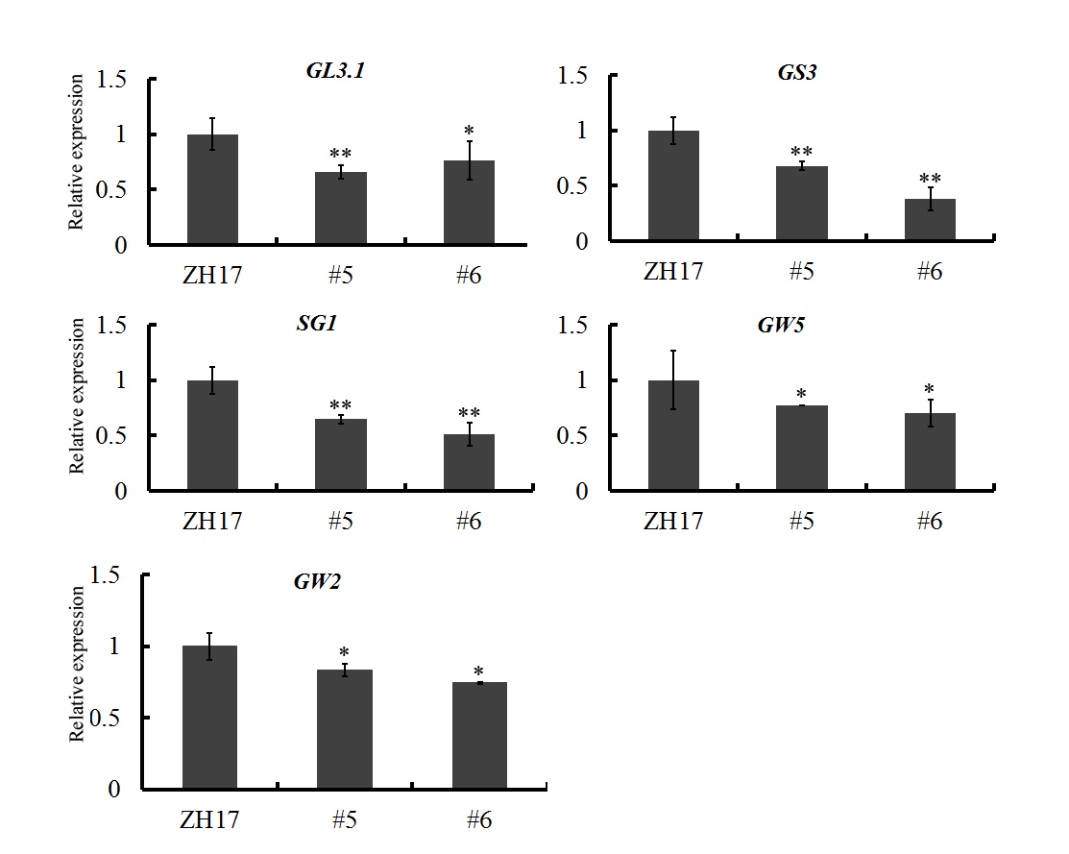
**
